# Crystal structures of two stilbazole derivatives: bis­{(*E*)-4-[4-(di­ethyl­amino)­styr­yl]-1-methyl­pyridin-1-ium} tetra­iodido­cadmium(II) and (*E*)-4-[4-(di­ethyl­amino)­styr­yl]-1-methyl­pyridin-1-ium 4-meth­oxy­benzene­sulfonate monohydrate

**DOI:** 10.1107/S2056989018016808

**Published:** 2018-11-30

**Authors:** Priya Antony, S. Antony Inglebert, Jerald V. Ramaclus, S. John Sundaram, P. Sagayaraj

**Affiliations:** a Department of Physics, Loyola College (Autonomous), Chennai - 600 034, India; b Department of Physics, St.Joseph’s College (Autonomous), Trichi - 600 002, India; c Department of Physics, Sacred Heart College (Autonomous), Tirupattur - 600 601, India

**Keywords:** crystal structure, stilbazole, 4-styryl­pyridine derivatives, tetra­iodo­cadmate, hydrogen bonding, ring motif, π-π inter­actions

## Abstract

The title mol­ecular salts, (C_18_H_23_N_2_)_2_[CdI_4_], (I), and C_18_H_23_N_2_^+^·C_7_H_7_O_4_S^−^·H_2_O, (II), are stilbazole, or 4-styryl­pyridine, derivatives. The cation, (*E*)-4-[4-(di­ethyl­amino)­styr­yl]-1-methyl­pyridin-1-ium, has a methyl group attached to pyridine ring and a diethyl amine group attached to the benzene ring. The asymmetric unit of salt (I), comprises one cationic mol­ecule and half a CdI_4_ dianion. The Cd atom is situated on a twofold rotation axis and has a slightly distorted tetra­hedral coordination sphere. In (II), the anion consists of a 4-meth­oxy­benzene­sulfonate and it crystallizes as a monohydrate. In both salts, the cations adopt an *E* configuration with respect to the C=C bond and the pyridine and benzene rings are inclined to each other by 10.7 (4)° in (I) and 4.6 (2)° in (II). In the crystals of both salts, the packing is consolidated by offset π–π stacking inter­actions involving the pyridinium and benzene rings, with centroid–centroid distances of 3.627 (4) Å in (I) and 3.614 (3) Å in (II). In the crystal of (II), a pair of 4-meth­oxy­benzene­sulfonate anions are bridged by O_water_—H⋯O_sulfonate_ hydrogen bonds, forming loops with an *R* ^2^_4_(8) motif. These four-membered units are then linked to the cations by a number of C—H⋯O hydrogen bonds, forming slabs lying parallel to the *ab* plane.

## Abstract 2

The title mol­ecular salts are stilbazole, or 4-styryl­pyridine, derivatives in which the cation has a methyl group attached to the pyridine ring N atom and a diethyl amine group attached to the benzene ring. In salt (I), the cadmium atom of the [CdI_4_]^2−^ dianion is located on a twofold rotation axis and the compound crystallizes with one cation in the asymmetric unit. In salt(II), the anion consists of a 4-meth­oxy­benzene­sulfonate ion, and it crystallizes as a monohydrate.

## Chemical context   

Stilbene-based compounds have been reported to possess a wide range of biological applications including anti­bacterial (Chanawanno *et al.*, 2010 ▸) and anti­oxidant (Frombaum *et al.*, 2012 ▸) activities. The anti­bacterial activities of a series of pyridine stilbene benzene­sulfonates have been studied against both gram-positive and gram-negative bacteria (Chanawanno *et al.*, 2010 ▸). Pyridine and its derivatives play an important role in drugs including anti­viral, anti­fungal, anti­bacterial, anti-inflammatory, anti­microbial, anti­cancer, anti­oxidant and anti­diabetic agents (Ghattas *et al.*, 2017 ▸). They have a variety of biological activities and a number of such compounds are in clinical use (Altaf *et al.*, 2015 ▸). The anti­bacterial activity of pyridinium derivatives have also been studied (Chanawanno *et al.*, 2010 ▸). The title salts, bis­[(*E*)-4-[4-(di­ethyl­amino)­styr­yl]-1-methyl­pyridin-1-ium] tetra­iodido­cadmate (I) and (*E*)-4-[4-(di­ethyl­amino)­styr­yl]-1-methyl­pyridin-1-ium 4-meth­oxy­benzene­sulfonate monohydrate (II) were tested for the level of cytotoxicity and anti­cancer analysis on normal VERO and MCF-7 cells. From an MTT assay it was found that the reported compounds have IC50 values of 31.2 µg mL^−1^ and 125 µg mL^−1^, respectively, against MCF-7 cell lines, whereas the IC50 value of crystals against normal VERO cells was found to be 1000 µg mL^−1^. This shows that both compounds exhibit very good anti­cancer activity, which implies that they may be suitable for biomedical applications.
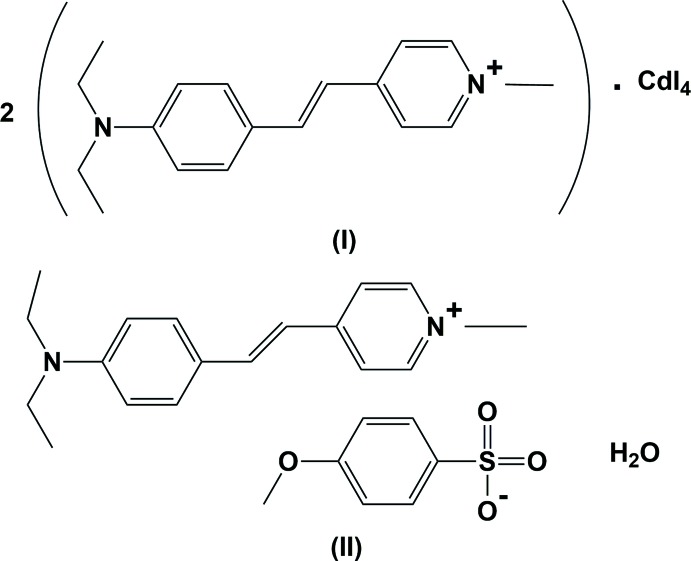


## Structural commentary   

**Figure 1:**
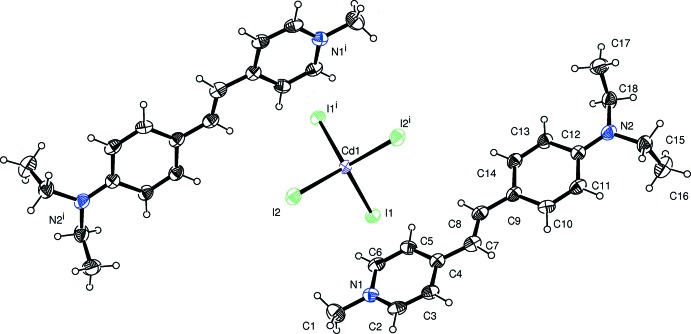
A view of the mol­ecular structure of salt (I), with the atom labelling. Displacement ellipsoids drawn at the 30% probability level. [symmetry code: (i) −*x*, *y*, −*z* + 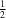.]

**Figure 2:**
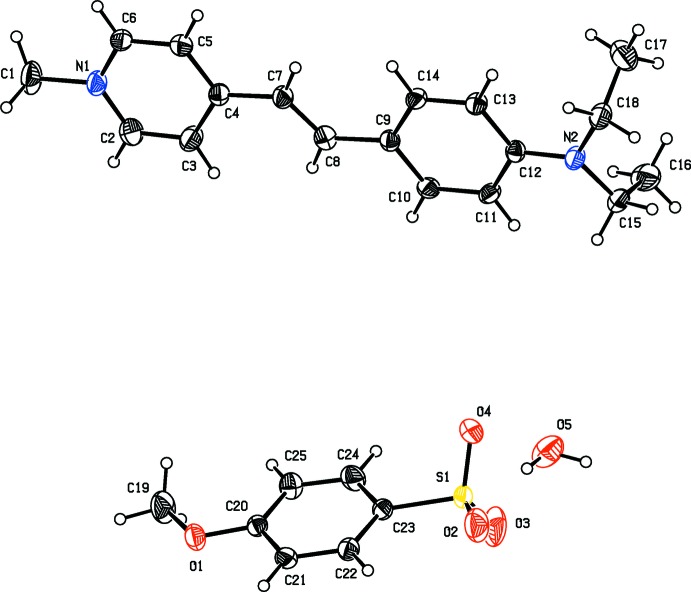
The mol­ecular structure of salt (II), with atom labelling. Displacement ellipsoids are drawn at the 30% probability level.

The title mol­ecular salts consist of the same cationic stilbazole derivative, (*E*)-4-[4-(di­ethyl­amino)­styr­yl]-1-methyl­pyridin-1-ium. Their mol­ecular structures are illustrated in Fig. 1 ▸ for (I) and Fig. 2 ▸ for (II). Salt (I) crystallizes with one 4-[4-(di­ethyl­amino)­styr­yl]-1-methyl­pyridin-1-ium cation and half a [CdI_4_]^2−^ anion in the asymmetric unit, the cadmium atom being located on a twofold rotation axis. The cadmium atom is surrounded by four iodine atoms with a slightly distorted tetra­hedral coordination sphere. In salt (II), the anion is 4-meth­oxy­benzene­sulfonate and it crystallizes as a monohydrate. In the cations of both salts, the configuration about the C7=C8 bond is *E*, with the C4—C7=C8—C9 torsion angle being 179.6 (6) ° in (I) and 178.7 (4)° in (II).

The dihedral angles between the mean planes of the pyridinium (N1/C2–C6) and benzene (C9–C14) rings are 10.7 (4) and 4.6 (2)° in (I) and (II), respectively. The C1—N1—C6—C5 torsion angles are −179.9 (7) and 179.1 (4)°, in (I) and (II), respectively, indicating that the methyl substituent (atom C1) at N1 is coplanar with the pyridine ring. The nitro­gen atom (N2) deviates from the benzene ring (C9–C14) plane by 0.023 (7) and 0.079 (3) Å in (I) and (II), respectively. The two ethyl units are orthogonal to the benzene ring, as indicated by torsion angle C17—C18—N2—C12, which is 89.1 (8)° in (I) and −81.7 (5)° in (II). The title salts exhibit structural similarities with related structures, as described in the *Database survey* below.

## Supra­molecular features   

**Figure 3:**
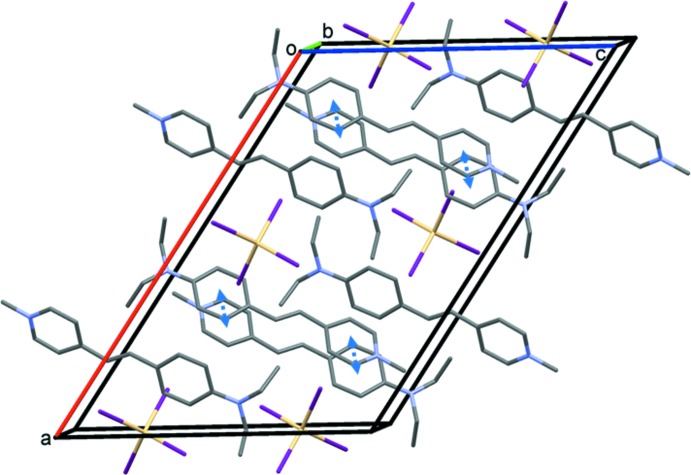
The crystal packing of salt (I), viewed along the *b* axis, showing the π–π inter­actions as double-headed blue arrows. For clarity, all of the H atoms have omitted.

In the crystal of (I), pairs of cations are arranged head-to-tail and the only significant inter­molecular inter­actions present are offset π–π inter­actions (Fig. 3 ▸). These involve the benzene (C9–C14; centroid *Cg*2) and pyridine (N1/C2–C6; centroid *Cg*1) rings [*Cg*2⋯*Cg*1^i^ = 3.627 (4) Å, α = 10.7 (4)°, β = 25.0°, inter­planar distances are 3.287 (3) and 3.503 (3) Å, offset = 0.941 Å, symmetry code: (i) −*x* + 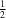, −*y* + 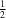, −*z* + 1].

**Figure 4:**
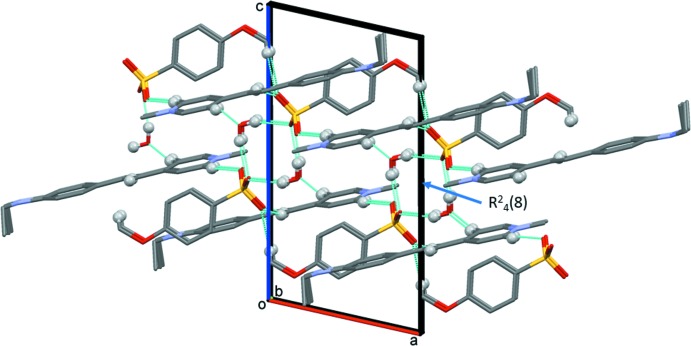
The crystal packing of salt (II), viewed along the *b*-axis, showing the hydrogen bonds (Table 1 ▸) as dashed lines. Only the H atoms (grey balls) involved in these inter­actions have been included.

In the crystal of (II), a pair of 4-meth­oxy­benzene­sulfonate anions are bridged by O_water_—H⋯O_sulfonate_ hydrogen bonds, forming loops with an 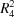
(8) graph-set motif (Table 1 ▸ and Fig. 4 ▸). These four-membered units are then linked to the cations by a number of C—H⋯O hydrogen bonds, forming slabs lying parallel to the *ab* plane (Table 1 ▸ and Fig. 4 ▸). Within the slabs there are offset π–π inter­actions present involving adjacent cations [*Cg*2⋯*Cg*1^ii^ = 3.614 (3) Å, α = 4.6 (2)°, β = 15.5°, inter­planar distances are 3.425 (2) and 3.484 (2) Å, offset = 0.963 Å, symmetry code: (ii) *x* − 1, *y*, *z*].

## Database survey   

A search of the Cambridge Structural Database (CSD, version 5.39, latest update August 2018; Groom *et al.*, 2016 ▸) for salts containing the title cation, 4-[4-(di­ethyl­amino)­styr­yl]-1-methyl­pyridin-1-ium, gave 12 hits; atomic coordinates are available for only 10 compounds. In the triiodide salt (CSD refcode EWUDUV; Tan *et al.*, 2004 ▸), the pyridinium and benzene rings are inclined to each other by *ca* 4.08°, while in the tetra­phenyl­borate salt (QECXON; Li *et al.*, 2012 ▸), the same dihedral angle is *ca* 14.33°, and in the iodide dihydrate salt (WOWGOE; Wang *et al.*, 2000 ▸) it is *ca* 8.77°. The corresponding dihedral angle in salt (I) is 10.7 (4)°. In the crystals of these compounds, π–π stacking inter­actions dominate, as in the crystal of (I).

There is only one salt reported with the title cation and a sulfonate anion, namely the *p*-toluene­sulfonate monohydrate salt (IBOWIG; Zhou *et al.*, 2004 ▸). Here the dihedral angle between the pyridinium and benzene rings in the cation is *ca* 6.88°, compared to 4.6 (2)° in salt (II). The crystal packing is very similar to that of salt (II): a pair of water mol­ecules bridge a pair of *p*-toluene­sulfonate anions via O—H⋯O hydrogen bonds, forming an 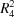
(8) ring motif; these four-membered units are linked to the cations by C—H⋯O hydrogen bonds, forming a network structure.

## Synthesis and crystallization   

**Compound (I)**

(*E*)-4-[4-(di­ethyl­amino)­styr­yl]-1-methyl-pyridinium-iodide (0.788 g, 2 mmol) and cadmium iodide (0.732 g, 2 mmol) were dissolved in a composite solvent, 2:1 ratio of aceto­nitrile and double-distilled water. The mixture was stirred well at 343 K and then allowed to cool naturally to room temperature. The solution was filtered and the filtrate left for the solvent to slowly evaporate at room temperature. After 3–4 weeks, dark-brown block-like crystals of compound (I) were obtained.

**Compound (II)**

(*E*)-4-[4-(di­ethyl­amino)­styr­yl]-1-methyl­pyridinium iodide (0.7885 g, 2 mmol) was mixed with sodium 4-meth­oxy­benzene­sulfonate (0.418 g, 2 mmol) in distilled water and heated at 373 K for 30 min. The mixture immediately yielded a grey precipitate of sodium iodide. After stirring the mixture for 30 min, the sodium iodide precipitate was removed. The filtrate was left to slowly evaporate and gave a deep-red solid. Red block-like crystals of compound (II), suitable for X-ray diffraction analysis, were obtained by slow evaporation of a solution in methanol after 2-3 weeks.

## Refinement   

Crystal data, data collection and structure refinement details for salts (I), and (II) are summarized in Table 2 ▸. The hydrogen atoms were located in difference electron-density maps. During refinement they were placed in idealized positions and allowed to ride on the parent atoms: C—H = 0.93–0.97Å with *U*_iso_(H) = 1.5*U*_eq_(C-meth­yl) and 1.2*U*_eq_(C,N) for other H atoms. The rotation angles for the methyl groups were optimized by least-squares. In compound (II), the hydrogen atoms of the water mol­ecule were treated as riding with *d*(O—H) = 0.85 Å and *U*_iso_(H) = 1.5*U*_eq_(O).

## Supplementary Material

Crystal structure: contains datablock(s) I, II, global. DOI: 10.1107/S2056989018016808/su5462sup1.cif

Structure factors: contains datablock(s) I. DOI: 10.1107/S2056989018016808/su5462Isup2.hkl

Structure factors: contains datablock(s) II. DOI: 10.1107/S2056989018016808/su5462IIsup3.hkl

Supporting information file. DOI: 10.1107/S2056989018016808/su5462IIsup4.cml

CCDC references: 1589674, 1589675

Additional supporting information:  crystallographic information; 3D view; checkCIF report

## Figures and Tables

**Table 1:** Hydrogen-bond geometry (Å, °) for (II)

*D*—H⋯*A*	*D*—H	H⋯*A*	*D*⋯*A*	*D*—H⋯*A*
O5—H5*A*⋯O3	0.85	2.06	2.891 (6)	165
O5—H5*B*⋯O3^i^	0.85	2.06	2.882 (6)	162
C3—H14⋯O5^ii^	0.93	2.49	3.394 (6)	163
C6—H17⋯O4^iii^	0.93	2.33	3.247 (6)	169
C7—H12⋯O2^iv^	0.93	2.58	3.476 (6)	162
C19—H19*A*⋯O2^ii^	0.96	2.53	3.423 (6)	155

Symmetry codes: (i) 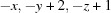; (ii) 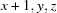; (iii) 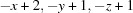; (iv) 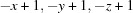.

**Table 2:** Experimental details

	(I)	(II)
Crystal data		
Chemical formula	(C_18_H_23_N_2_)_2_[CdI_4_]	C_18_H_23_N_2_^+^·C_7_H_7_O_4_S^−^·H_2_O
*M*_r_	1154.77	472.59
Crystal system, space group	Monoclinic, *C*2/*c*	Triclinic, *P* 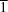
Temperature (K)	296	296
*a*, *b*, *c* (Å)	21.6649 (18), 14.9748 (12), 14.9744 (11)	8.2481 (6), 9.7963 (9), 15.5409 (14)
α, β, γ (°)	90, 123.621 (2), 90	94.283 (5), 101.647 (5), 99.112 (5)
*V* (Å^3^)	4045.4 (6)	1206.93 (18)
*Z*	4	2
Radiation type	Mo *K*α	Mo *K*α
μ (mm^−1^)	3.62	0.17
Crystal size (mm)	0.15 × 0.15 × 0.10	0.38 × 0.30 × 0.18

Data collection		
Diffractometer	Bruker Kappa APEXII CCD	Bruker Kappa APEXII CCD
Absorption correction	Multi-scan (*SADABS*; Bruker, 2008 ▸)	Multi-scan (*SADABS*; Bruker, 2008 ▸)
*T*_min_, *T*_max_	0.613, 0.713	0.940, 0.969
No. of measured, independent and observed [*I* > 2σ(*I*)] reflections	37329, 5003, 2802	25709, 4253, 2396
*R*_int_	0.070	0.167
(sin θ/λ)_max_ (Å^−1^)	0.666	0.595

Refinement		
*R*[*F* ^2^ > 2σ(*F* ^2^)], *wR*(*F* ^2^), *S*	0.043, 0.091, 1.02	0.080, 0.161, 1.07
No. of reflections	5003	4253
No. of parameters	207	306
H-atom treatment	H atoms treated by a mixture of independent and constrained refinement	H atoms treated by a mixture of independent and constrained refinement
Δρ_max_, Δρ_min_ (e Å^−3^)	1.23, −0.87	0.30, −0.22

Computer programs: *APEX2* and *SAINT* (Bruker, 2008 ▸), *SHELXS97* and *SHELXL97* (Sheldrick, 2008 ▸), *ORTEP-3 for Windows* (Farrugia, 2012 ▸), *Mercury* (Macrae *et al.*, 2008 ▸) and *PLATON* (Spek, 2009 ▸).

## supplementary crystallographic information

### Bis{(E)-4-[4-(diethylamino)styryl]-1-methylpyridin-1-ium} tetraiodidocadmium(II) (I) Crystal data

**Table:**

(C_18_H_23_N_2_)_2_[CdI_4_]	*F*(000) = 2200
*M**_r_* = 1154.77	*D*_x_ = 1.896 Mg m^−^^3^
Monoclinic, *C*2/*c*	Mo *K*α radiation, λ = 0.71073 Å
Hall symbol: -C 2 y c	Cell parameters from 5003 reflections
*a* = 21.6649 (18) Å	θ = 1.9–28.3°
*b* = 14.9748 (12) Å	µ = 3.62 mm^−^^1^
*c* = 14.9744 (11) Å	*T* = 296 K
β = 123.621 (2)°	Block, brown
*V* = 4045.4 (6) Å^3^	0.15 × 0.15 × 0.10 mm
*Z* = 4	

### Bis{(E)-4-[4-(diethylamino)styryl]-1-methylpyridin-1-ium} tetraiodidocadmium(II) (I) Data collection

**Table:**

Bruker Kappa APEXII CCD diffractometer	5003 independent reflections
Radiation source: fine-focus sealed tube	2802 reflections with *I* > 2σ(*I*)
Graphite monochromator	*R*_int_ = 0.070
ω and φ scan	θ_max_ = 28.3°, θ_min_ = 1.9°
Absorption correction: multi-scan (SADABS; Bruker, 2008)	*h* = −28→28
*T*_min_ = 0.613, *T*_max_ = 0.713	*k* = −19→19
37329 measured reflections	*l* = −19→19

### Bis{(E)-4-[4-(diethylamino)styryl]-1-methylpyridin-1-ium} tetraiodidocadmium(II) (I) Refinement

**Table:**

Refinement on *F*^2^	Primary atom site location: structure-invariant direct methods
Least-squares matrix: full	Secondary atom site location: difference Fourier map
*R*[*F*^2^ > 2σ(*F*^2^)] = 0.043	Hydrogen site location: inferred from neighbouring sites
*wR*(*F*^2^) = 0.091	H atoms treated by a mixture of independent and constrained refinement
*S* = 1.02	*w* = 1/[σ^2^(*F*_o_^2^) + (0.0128*P*)^2^ + 39.5734*P*] where *P* = (*F*_o_^2^ + 2*F*_c_^2^)/3
5003 reflections	(Δ/σ)_max_ = 0.001
207 parameters	Δρ_max_ = 1.23 e Å^−^^3^
0 restraints	Δρ_min_ = −0.87 e Å^−^^3^

### Bis{(E)-4-[4-(diethylamino)styryl]-1-methylpyridin-1-ium} tetraiodidocadmium(II) (I) Special details

**Table:**

Geometry. All esds (except the esd in the dihedral angle between two l.s. planes) are estimated using the full covariance matrix. The cell esds are taken into account individually in the estimation of esds in distances, angles and torsion angles; correlations between esds in cell parameters are only used when they are defined by crystal symmetry. An approximate (isotropic) treatment of cell esds is used for estimating esds involving l.s. planes.
Refinement. Refinement of F^2^ against ALL reflections. The weighted R-factor wR and goodness of fit S are based on F^2^, conventional R-factors R are based on F, with F set to zero for negative F^2^. The threshold expression of F^2^ > 2sigma(F^2^) is used only for calculating R-factors(gt) etc. and is not relevant to the choice of reflections for refinement. R-factors based on F^2^ are statistically about twice as large as those based on F, and R- factors based on ALL data will be even larger.

### Bis{(E)-4-[4-(diethylamino)styryl]-1-methylpyridin-1-ium} tetraiodidocadmium(II) (I) Fractional atomic coordinates and isotropic or equivalent isotropic displacement parameters (Å^2^)

**Table:**

	*x*	*y*	*z*	*U*_iso_*/*U*_eq_	
C1	0.1450 (5)	0.3509 (5)	0.0481 (6)	0.075 (2)	
H1A	0.1669	0.3954	0.0279	0.112*	
H1B	0.0941	0.3660	0.0187	0.112*	
H1C	0.1474	0.2938	0.0210	0.112*	
C2	0.2429 (4)	0.2922 (5)	0.2212 (6)	0.0576 (19)	
H2	0.2568	0.2556	0.1849	0.069*	
C3	0.2816 (4)	0.2883 (4)	0.3292 (6)	0.0539 (18)	
H3	0.3221	0.2501	0.3657	0.065*	
C4	0.2626 (4)	0.3397 (4)	0.3867 (5)	0.0456 (16)	
C5	0.2005 (4)	0.3962 (5)	0.3255 (6)	0.0563 (18)	
H5	0.1847	0.4320	0.3598	0.068*	
C6	0.1639 (4)	0.3987 (5)	0.2171 (6)	0.0568 (18)	
H6	0.1234	0.4365	0.1779	0.068*	
C7	0.3041 (4)	0.3361 (4)	0.5024 (6)	0.0536 (18)	
H7	0.3467	0.3011	0.5376	0.064*	
C8	0.2859 (4)	0.3792 (4)	0.5622 (6)	0.0533 (18)	
H8	0.2433	0.4139	0.5247	0.064*	
C9	0.3236 (4)	0.3796 (4)	0.6780 (6)	0.0509 (17)	
C10	0.3910 (4)	0.3350 (5)	0.7473 (6)	0.0554 (18)	
H10	0.4143	0.3050	0.7193	0.067*	
C11	0.4228 (4)	0.3355 (5)	0.8562 (6)	0.0575 (19)	
H11	0.4678	0.3063	0.9005	0.069*	
C12	0.3896 (4)	0.3791 (4)	0.9033 (5)	0.0476 (16)	
C13	0.3215 (4)	0.4221 (4)	0.8322 (6)	0.0534 (18)	
H13	0.2971	0.4509	0.8592	0.064*	
C14	0.2905 (4)	0.4221 (5)	0.7236 (6)	0.0549 (18)	
H14	0.2458	0.4517	0.6790	0.066*	
C15	0.4916 (4)	0.3359 (5)	1.0878 (6)	0.065 (2)	
H15A	0.4971	0.2833	1.0550	0.078*	
H15B	0.4927	0.3168	1.1506	0.078*	
C16	0.5558 (5)	0.3987 (6)	1.1222 (7)	0.094 (3)	
H16A	0.5564	0.4153	1.0608	0.142*	
H16B	0.6014	0.3694	1.1738	0.142*	
H16C	0.5501	0.4512	1.1537	0.142*	
C17	0.3302 (4)	0.3824 (6)	1.0656 (7)	0.080 (2)	
H17A	0.2889	0.3694	0.9943	0.120*	
H17B	0.3141	0.4196	1.1011	0.120*	
H17C	0.3501	0.3278	1.1049	0.120*	
C18	0.3893 (4)	0.4305 (5)	1.0603 (6)	0.066 (2)	
H18A	0.3684	0.4851	1.0194	0.079*	
H18B	0.4292	0.4470	1.1324	0.079*	
N1	0.1855 (3)	0.3468 (4)	0.1655 (5)	0.0519 (14)	
N2	0.4199 (3)	0.3780 (4)	1.0116 (5)	0.0603 (16)	
Cd1	0.0000	0.51199 (4)	0.2500	0.04358 (18)	
I1	0.10734 (3)	0.62756 (3)	0.26682 (4)	0.05415 (14)	
I2	−0.05786 (3)	0.40088 (4)	0.07160 (4)	0.06612 (17)	

### Bis{(E)-4-[4-(diethylamino)styryl]-1-methylpyridin-1-ium} tetraiodidocadmium(II) (I) Atomic displacement parameters (Å^2^)

**Table:**

	*U*^11^	*U*^22^	*U*^33^	*U*^12^	*U*^13^	*U*^23^
C1	0.095 (6)	0.063 (5)	0.070 (5)	−0.007 (5)	0.047 (5)	−0.014 (4)
C2	0.063 (5)	0.042 (4)	0.085 (6)	0.008 (4)	0.052 (5)	−0.003 (4)
C3	0.056 (4)	0.042 (4)	0.073 (5)	0.006 (3)	0.041 (4)	0.004 (4)
C4	0.049 (4)	0.029 (3)	0.061 (4)	−0.005 (3)	0.031 (4)	0.003 (3)
C5	0.063 (5)	0.056 (5)	0.061 (5)	0.004 (4)	0.041 (4)	−0.008 (4)
C6	0.053 (4)	0.049 (4)	0.078 (5)	0.004 (3)	0.042 (4)	−0.002 (4)
C7	0.057 (5)	0.033 (4)	0.076 (5)	0.003 (3)	0.041 (4)	0.006 (3)
C8	0.048 (4)	0.044 (4)	0.071 (5)	−0.004 (3)	0.035 (4)	0.006 (4)
C9	0.043 (4)	0.043 (4)	0.067 (5)	0.000 (3)	0.031 (4)	0.009 (3)
C10	0.055 (5)	0.055 (5)	0.070 (5)	−0.001 (4)	0.043 (4)	−0.003 (4)
C11	0.048 (4)	0.050 (4)	0.077 (5)	0.008 (3)	0.036 (4)	0.004 (4)
C12	0.046 (4)	0.041 (4)	0.054 (4)	0.000 (3)	0.027 (4)	0.002 (3)
C13	0.050 (4)	0.046 (4)	0.063 (5)	0.012 (3)	0.031 (4)	0.009 (3)
C14	0.048 (4)	0.049 (4)	0.064 (5)	0.007 (3)	0.028 (4)	0.009 (4)
C15	0.058 (5)	0.062 (5)	0.064 (5)	0.017 (4)	0.027 (4)	0.013 (4)
C16	0.060 (6)	0.096 (7)	0.104 (7)	0.001 (5)	0.031 (5)	0.004 (6)
C17	0.070 (6)	0.091 (7)	0.091 (6)	0.004 (5)	0.052 (5)	0.004 (5)
C18	0.061 (5)	0.067 (5)	0.057 (5)	0.012 (4)	0.025 (4)	−0.004 (4)
N1	0.056 (4)	0.045 (4)	0.061 (4)	−0.008 (3)	0.037 (3)	−0.006 (3)
N2	0.049 (4)	0.067 (4)	0.062 (4)	0.011 (3)	0.030 (3)	−0.001 (3)
Cd1	0.0366 (4)	0.0423 (4)	0.0472 (4)	0.000	0.0202 (3)	0.000
I1	0.0507 (3)	0.0489 (3)	0.0651 (3)	−0.0079 (2)	0.0335 (2)	−0.0002 (2)
I2	0.0539 (3)	0.0672 (4)	0.0688 (3)	−0.0091 (3)	0.0287 (3)	−0.0258 (3)

### Bis{(E)-4-[4-(diethylamino)styryl]-1-methylpyridin-1-ium} tetraiodidocadmium(II) (I) Geometric parameters (Å, º)

**Table:**

C1—N1	1.468 (9)	C11—H11	0.9300
C1—H1A	0.9600	C12—N2	1.371 (8)
C1—H1B	0.9600	C12—C13	1.409 (9)
C1—H1C	0.9600	C13—C14	1.373 (9)
C2—N1	1.328 (8)	C13—H13	0.9300
C2—C3	1.349 (9)	C14—H14	0.9300
C2—H2	0.9300	C15—N2	1.467 (8)
C3—C4	1.377 (9)	C15—C16	1.513 (10)
C3—H3	0.9300	C15—H15A	0.9700
C4—C5	1.414 (9)	C15—H15B	0.9700
C4—C7	1.445 (9)	C16—H16A	0.9600
C5—C6	1.356 (9)	C16—H16B	0.9600
C5—H5	0.9300	C16—H16C	0.9600
C6—N1	1.349 (8)	C17—C18	1.508 (10)
C6—H6	0.9300	C17—H17A	0.9600
C7—C8	1.328 (9)	C17—H17B	0.9600
C7—H7	0.9300	C17—H17C	0.9600
C8—C9	1.452 (9)	C18—N2	1.458 (9)
C8—H8	0.9300	C18—H18A	0.9700
C9—C14	1.388 (9)	C18—H18B	0.9700
C9—C10	1.407 (9)	Cd1—I2^i^	2.7871 (6)
C10—C11	1.374 (9)	Cd1—I2	2.7871 (6)
C10—H10	0.9300	Cd1—I1^i^	2.7960 (6)
C11—C12	1.416 (9)	Cd1—I1	2.7961 (6)
			
N1—C1—H1A	109.5	C12—C13—H13	119.5
N1—C1—H1B	109.5	C13—C14—C9	122.4 (7)
H1A—C1—H1B	109.5	C13—C14—H14	118.8
N1—C1—H1C	109.5	C9—C14—H14	118.8
H1A—C1—H1C	109.5	N2—C15—C16	112.0 (6)
H1B—C1—H1C	109.5	N2—C15—H15A	109.2
N1—C2—C3	121.6 (7)	C16—C15—H15A	109.2
N1—C2—H2	119.2	N2—C15—H15B	109.2
C3—C2—H2	119.2	C16—C15—H15B	109.2
C2—C3—C4	121.5 (7)	H15A—C15—H15B	107.9
C2—C3—H3	119.2	C15—C16—H16A	109.5
C4—C3—H3	119.2	C15—C16—H16B	109.5
C3—C4—C5	115.8 (6)	H16A—C16—H16B	109.5
C3—C4—C7	121.7 (6)	C15—C16—H16C	109.5
C5—C4—C7	122.5 (6)	H16A—C16—H16C	109.5
C6—C5—C4	120.6 (6)	H16B—C16—H16C	109.5
C6—C5—H5	119.7	C18—C17—H17A	109.5
C4—C5—H5	119.7	C18—C17—H17B	109.5
N1—C6—C5	120.8 (7)	H17A—C17—H17B	109.5
N1—C6—H6	119.6	C18—C17—H17C	109.5
C5—C6—H6	119.6	H17A—C17—H17C	109.5
C8—C7—C4	124.7 (7)	H17B—C17—H17C	109.5
C8—C7—H7	117.6	N2—C18—C17	113.8 (6)
C4—C7—H7	117.6	N2—C18—H18A	108.8
C7—C8—C9	128.8 (7)	C17—C18—H18A	108.8
C7—C8—H8	115.6	N2—C18—H18B	108.8
C9—C8—H8	115.6	C17—C18—H18B	108.8
C14—C9—C10	117.5 (7)	H18A—C18—H18B	107.7
C14—C9—C8	119.3 (6)	C2—N1—C6	119.8 (6)
C10—C9—C8	123.2 (7)	C2—N1—C1	120.6 (6)
C11—C10—C9	120.4 (7)	C6—N1—C1	119.6 (6)
C11—C10—H10	119.8	C12—N2—C18	122.2 (6)
C9—C10—H10	119.8	C12—N2—C15	122.2 (6)
C10—C11—C12	122.3 (7)	C18—N2—C15	114.9 (6)
C10—C11—H11	118.8	I2^i^—Cd1—I2	106.69 (3)
C12—C11—H11	118.8	I2^i^—Cd1—I1^i^	111.478 (16)
N2—C12—C13	121.0 (6)	I2—Cd1—I1^i^	111.895 (15)
N2—C12—C11	122.7 (6)	I2^i^—Cd1—I1	111.894 (15)
C13—C12—C11	116.3 (6)	I2—Cd1—I1	111.476 (16)
C14—C13—C12	121.0 (7)	I1^i^—Cd1—I1	103.52 (3)
C14—C13—H13	119.5		
			
N1—C2—C3—C4	1.3 (11)	C11—C12—C13—C14	−0.9 (10)
C2—C3—C4—C5	−0.2 (10)	C12—C13—C14—C9	0.7 (11)
C2—C3—C4—C7	−179.6 (6)	C10—C9—C14—C13	0.3 (10)
C3—C4—C5—C6	−0.7 (10)	C8—C9—C14—C13	177.4 (6)
C7—C4—C5—C6	178.8 (6)	C3—C2—N1—C6	−1.5 (10)
C4—C5—C6—N1	0.5 (11)	C3—C2—N1—C1	179.0 (7)
C3—C4—C7—C8	−175.3 (6)	C5—C6—N1—C2	0.6 (10)
C5—C4—C7—C8	5.3 (10)	C5—C6—N1—C1	−179.9 (7)
C4—C7—C8—C9	179.6 (6)	C13—C12—N2—C18	−8.3 (10)
C7—C8—C9—C14	−173.2 (7)	C11—C12—N2—C18	173.6 (7)
C7—C8—C9—C10	3.7 (11)	C13—C12—N2—C15	−178.4 (6)
C14—C9—C10—C11	−1.1 (10)	C11—C12—N2—C15	3.6 (10)
C8—C9—C10—C11	−178.0 (6)	C17—C18—N2—C12	89.1 (8)
C9—C10—C11—C12	0.9 (11)	C17—C18—N2—C15	−100.2 (8)
C10—C11—C12—N2	178.3 (7)	C16—C15—N2—C12	85.9 (9)
C10—C11—C12—C13	0.1 (10)	C16—C15—N2—C18	−84.8 (8)
N2—C12—C13—C14	−179.1 (7)		

Symmetry code: (i) −*x*, *y*, −*z*+1/2.

### 4-{2-[4-(Diethylamino)phenyl]ethenyl}-1-methylpyridin-1-ium 4-methoxybenzene-1-sulfonate monohydrate (II) Crystal data

**Table:**

C_18_H_23_N_2_^+^·C_7_H_7_O_4_S^−^·H_2_O	*Z* = 2
*M**_r_* = 472.59	*F*(000) = 504
Triclinic, *P*1	*D*_x_ = 1.300 Mg m^−^^3^
Hall symbol: -P1	Mo *K*α radiation, λ = 0.71073 Å
*a* = 8.2481 (6) Å	Cell parameters from 4253 reflections
*b* = 9.7963 (9) Å	θ = 3.0–25.0°
*c* = 15.5409 (14) Å	µ = 0.17 mm^−^^1^
α = 94.283 (5)°	*T* = 296 K
β = 101.647 (5)°	Block, red
γ = 99.112 (5)°	0.38 × 0.30 × 0.18 mm
*V* = 1206.93 (18) Å^3^	

### 4-{2-[4-(Diethylamino)phenyl]ethenyl}-1-methylpyridin-1-ium 4-methoxybenzene-1-sulfonate monohydrate (II) Data collection

**Table:**

Bruker Kappa APEXII CCD diffractometer	4253 independent reflections
Radiation source: fine-focus sealed tube	2396 reflections with *I* > 2σ(*I*)
Graphite monochromator	*R*_int_ = 0.167
ω and φ scan	θ_max_ = 25.0°, θ_min_ = 3.0°
Absorption correction: multi-scan (SADABS; Bruker, 2008)	*h* = −9→9
*T*_min_ = 0.940, *T*_max_ = 0.969	*k* = −11→11
25709 measured reflections	*l* = −18→18

### 4-{2-[4-(Diethylamino)phenyl]ethenyl}-1-methylpyridin-1-ium 4-methoxybenzene-1-sulfonate monohydrate (II) Refinement

**Table:**

Refinement on *F*^2^	Secondary atom site location: difference Fourier map
Least-squares matrix: full	Hydrogen site location: inferred from neighbouring sites
*R*[*F*^2^ > 2σ(*F*^2^)] = 0.080	H atoms treated by a mixture of independent and constrained refinement
*wR*(*F*^2^) = 0.161	*w* = 1/[σ^2^(*F*_o_^2^) + (0.0418*P*)^2^ + 1.3526*P*] where *P* = (*F*_o_^2^ + 2*F*_c_^2^)/3
*S* = 1.07	(Δ/σ)_max_ < 0.001
4253 reflections	Δρ_max_ = 0.30 e Å^−^^3^
306 parameters	Δρ_min_ = −0.22 e Å^−^^3^
0 restraints	Extinction correction: SHELXL97 (Sheldrick, 2008), Fc^*^=kFc[1+0.001xFc^2^λ^3^/sin(2θ)]^-1/4^
Primary atom site location: structure-invariant direct methods	Extinction coefficient: 0.0075 (14)

### 4-{2-[4-(Diethylamino)phenyl]ethenyl}-1-methylpyridin-1-ium 4-methoxybenzene-1-sulfonate monohydrate (II) Special details

**Table:**

Geometry. All esds (except the esd in the dihedral angle between two l.s. planes) are estimated using the full covariance matrix. The cell esds are taken into account individually in the estimation of esds in distances, angles and torsion angles; correlations between esds in cell parameters are only used when they are defined by crystal symmetry. An approximate (isotropic) treatment of cell esds is used for estimating esds involving l.s. planes.
Refinement. Refinement of F^2^ against ALL reflections. The weighted R-factor wR and goodness of fit S are based on F^2^, conventional R-factors R are based on F, with F set to zero for negative F^2^. The threshold expression of F^2^ > 2sigma(F^2^) is used only for calculating R-factors(gt) etc. and is not relevant to the choice of reflections for refinement. R-factors based on F^2^ are statistically about twice as large as those based on F, and R- factors based on ALL data will be even larger.

### 4-{2-[4-(Diethylamino)phenyl]ethenyl}-1-methylpyridin-1-ium 4-methoxybenzene-1-sulfonate monohydrate (II) Fractional atomic coordinates and isotropic or equivalent isotropic displacement parameters (Å^2^)

**Table:**

	*x*	*y*	*z*	*U*_iso_*/*U*_eq_	
C1	1.8223 (5)	0.6523 (6)	0.4728 (3)	0.0666 (16)	
H16A	1.8691	0.5725	0.4575	0.100*	
H16B	1.8368	0.6672	0.5359	0.100*	
H16C	1.8788	0.7327	0.4522	0.100*	
C2	1.5563 (5)	0.7352 (5)	0.4278 (3)	0.0565 (14)	
H15	1.6137	0.8236	0.4519	0.068*	
C3	1.3901 (5)	0.7177 (5)	0.3904 (3)	0.0505 (13)	
H14	1.3355	0.7938	0.3893	0.061*	
C4	1.3003 (5)	0.5865 (5)	0.3536 (3)	0.0360 (11)	
C5	1.3915 (5)	0.4783 (5)	0.3598 (3)	0.0444 (12)	
H18	1.3367	0.3883	0.3379	0.053*	
C6	1.5597 (5)	0.5008 (5)	0.3973 (3)	0.0466 (12)	
H17	1.6180	0.4268	0.3994	0.056*	
C7	1.1240 (5)	0.5583 (5)	0.3109 (3)	0.0409 (11)	
H12	1.0741	0.4656	0.2943	0.049*	
C8	1.0267 (5)	0.6531 (5)	0.2931 (3)	0.0400 (11)	
H11	1.0770	0.7455	0.3109	0.048*	
C9	0.8504 (5)	0.6276 (5)	0.2488 (3)	0.0346 (10)	
C10	0.7625 (5)	0.7379 (4)	0.2380 (3)	0.0392 (11)	
H9	0.8197	0.8277	0.2585	0.047*	
C11	0.5938 (5)	0.7186 (4)	0.1981 (3)	0.0377 (11)	
H6	0.5404	0.7951	0.1914	0.045*	
C12	0.5027 (5)	0.5848 (4)	0.1678 (3)	0.0342 (10)	
C13	0.5919 (5)	0.4738 (4)	0.1788 (3)	0.0375 (11)	
H7	0.5354	0.3835	0.1592	0.045*	
C14	0.7586 (5)	0.4953 (4)	0.2173 (3)	0.0368 (11)	
H8	0.8130	0.4192	0.2228	0.044*	
C15	0.2459 (5)	0.6762 (5)	0.1087 (3)	0.0469 (12)	
H2A	0.2845	0.7525	0.1557	0.056*	
H2B	0.1265	0.6458	0.1044	0.056*	
C16	0.2708 (7)	0.7292 (5)	0.0234 (3)	0.0673 (15)	
H1A	0.3878	0.7656	0.0282	0.101*	
H1B	0.2063	0.8016	0.0109	0.101*	
H1C	0.2343	0.6545	−0.0236	0.101*	
C17	0.2467 (6)	0.3604 (5)	0.0164 (3)	0.0712 (16)	
H3A	0.1996	0.4155	−0.0275	0.107*	
H3B	0.1848	0.2669	0.0045	0.107*	
H3C	0.3622	0.3598	0.0146	0.107*	
C18	0.2363 (5)	0.4213 (5)	0.1064 (3)	0.0510 (13)	
H4A	0.1193	0.4231	0.1070	0.061*	
H4B	0.2765	0.3610	0.1496	0.061*	
C19	0.9837 (5)	0.9933 (6)	0.8722 (3)	0.0708 (17)	
H19A	0.9936	0.9136	0.8351	0.106*	
H19B	1.0760	1.0114	0.9227	0.106*	
H19C	0.9854	1.0726	0.8395	0.106*	
C20	0.6835 (5)	0.9375 (4)	0.8384 (3)	0.0351 (10)	
C21	0.5389 (5)	0.9182 (4)	0.8707 (3)	0.0396 (11)	
H21	0.5454	0.9266	0.9313	0.048*	
C22	0.3847 (5)	0.8864 (4)	0.8129 (3)	0.0393 (11)	
H22	0.2873	0.8715	0.8349	0.047*	
C23	0.3731 (5)	0.8764 (4)	0.7233 (3)	0.0348 (10)	
C24	0.5190 (6)	0.8983 (5)	0.6920 (3)	0.0495 (12)	
H24	0.5121	0.8918	0.6313	0.059*	
C25	0.6738 (5)	0.9292 (5)	0.7482 (3)	0.0467 (12)	
H25	0.7710	0.9444	0.7261	0.056*	
N1	1.6404 (4)	0.6282 (4)	0.4310 (2)	0.0452 (10)	
N2	0.3330 (4)	0.5618 (4)	0.1330 (2)	0.0407 (9)	
O1	0.8308 (3)	0.9673 (3)	0.90069 (18)	0.0489 (8)	
O2	0.0530 (4)	0.7905 (4)	0.6988 (2)	0.0754 (11)	
O3	0.1484 (4)	0.9579 (4)	0.6101 (3)	0.0907 (14)	
O4	0.1904 (4)	0.7254 (4)	0.5852 (2)	0.0862 (13)	
O5	0.1888 (5)	0.9840 (5)	0.4312 (3)	0.0791 (12)	
H5A	0.1915	0.9678	0.4844	0.119*	
H5B	0.0903	0.9951	0.4070	0.119*	
S1	0.17473 (14)	0.83367 (13)	0.64771 (8)	0.0441 (4)	

### 4-{2-[4-(Diethylamino)phenyl]ethenyl}-1-methylpyridin-1-ium 4-methoxybenzene-1-sulfonate monohydrate (II) Atomic displacement parameters (Å^2^)

**Table:**

	*U*^11^	*U*^22^	*U*^33^	*U*^12^	*U*^13^	*U*^23^
C1	0.031 (2)	0.110 (5)	0.057 (3)	0.013 (3)	0.004 (2)	0.013 (3)
C2	0.040 (3)	0.057 (4)	0.064 (3)	0.000 (2)	0.003 (2)	−0.004 (3)
C3	0.038 (3)	0.047 (3)	0.066 (3)	0.012 (2)	0.007 (2)	0.005 (3)
C4	0.031 (2)	0.049 (3)	0.031 (2)	0.008 (2)	0.0116 (19)	0.006 (2)
C5	0.041 (3)	0.050 (3)	0.040 (3)	0.010 (2)	0.008 (2)	−0.003 (2)
C6	0.043 (3)	0.068 (4)	0.032 (3)	0.023 (3)	0.009 (2)	−0.002 (2)
C7	0.031 (2)	0.053 (3)	0.038 (3)	0.008 (2)	0.0069 (19)	0.001 (2)
C8	0.037 (2)	0.046 (3)	0.038 (3)	0.002 (2)	0.011 (2)	0.007 (2)
C9	0.032 (2)	0.043 (3)	0.029 (2)	0.007 (2)	0.0075 (18)	0.004 (2)
C10	0.039 (2)	0.029 (3)	0.046 (3)	0.002 (2)	0.007 (2)	−0.002 (2)
C11	0.036 (2)	0.032 (3)	0.047 (3)	0.013 (2)	0.009 (2)	0.004 (2)
C12	0.033 (2)	0.038 (3)	0.032 (2)	0.007 (2)	0.0063 (18)	0.007 (2)
C13	0.037 (2)	0.028 (3)	0.045 (3)	0.0047 (19)	0.006 (2)	0.006 (2)
C14	0.039 (2)	0.037 (3)	0.037 (3)	0.014 (2)	0.007 (2)	0.006 (2)
C15	0.037 (2)	0.049 (3)	0.056 (3)	0.016 (2)	0.007 (2)	0.003 (3)
C16	0.091 (4)	0.057 (4)	0.060 (4)	0.033 (3)	0.013 (3)	0.015 (3)
C17	0.080 (4)	0.060 (4)	0.060 (4)	0.005 (3)	−0.007 (3)	0.000 (3)
C18	0.040 (3)	0.048 (3)	0.062 (3)	0.011 (2)	0.003 (2)	0.007 (3)
C19	0.035 (3)	0.104 (5)	0.066 (4)	0.000 (3)	0.007 (2)	−0.003 (3)
C20	0.037 (2)	0.030 (3)	0.037 (3)	0.0075 (19)	0.004 (2)	0.006 (2)
C21	0.044 (3)	0.041 (3)	0.034 (3)	0.010 (2)	0.008 (2)	0.002 (2)
C22	0.035 (2)	0.039 (3)	0.043 (3)	0.007 (2)	0.007 (2)	0.005 (2)
C23	0.041 (2)	0.029 (3)	0.035 (3)	0.0110 (19)	0.0020 (19)	0.006 (2)
C24	0.060 (3)	0.058 (3)	0.032 (3)	0.012 (2)	0.009 (2)	0.008 (2)
C25	0.041 (3)	0.053 (3)	0.046 (3)	0.004 (2)	0.010 (2)	0.010 (2)
N1	0.0282 (19)	0.066 (3)	0.041 (2)	0.011 (2)	0.0063 (17)	0.003 (2)
N2	0.0310 (18)	0.033 (2)	0.055 (2)	0.0067 (16)	0.0003 (16)	0.0042 (18)
O1	0.0372 (17)	0.060 (2)	0.0427 (19)	0.0038 (15)	−0.0012 (14)	0.0009 (16)
O2	0.0422 (19)	0.102 (3)	0.073 (3)	0.0049 (19)	−0.0002 (18)	0.002 (2)
O3	0.084 (3)	0.063 (3)	0.108 (3)	0.019 (2)	−0.032 (2)	0.032 (2)
O4	0.057 (2)	0.106 (3)	0.078 (3)	0.031 (2)	−0.0189 (18)	−0.047 (2)
O5	0.071 (2)	0.094 (3)	0.088 (3)	0.035 (2)	0.032 (2)	0.025 (3)
S1	0.0407 (7)	0.0404 (8)	0.0454 (7)	0.0147 (5)	−0.0075 (5)	−0.0015 (6)

### 4-{2-[4-(Diethylamino)phenyl]ethenyl}-1-methylpyridin-1-ium 4-methoxybenzene-1-sulfonate monohydrate (II) Geometric parameters (Å, º)

**Table:**

C1—N1	1.484 (5)	C15—H2B	0.9700
C1—H16A	0.9600	C16—H1A	0.9600
C1—H16B	0.9600	C16—H1B	0.9600
C1—H16C	0.9600	C16—H1C	0.9600
C2—N1	1.344 (6)	C17—C18	1.503 (6)
C2—C3	1.354 (6)	C17—H3A	0.9600
C2—H15	0.9300	C17—H3B	0.9600
C3—C4	1.395 (6)	C17—H3C	0.9600
C3—H14	0.9300	C18—N2	1.461 (5)
C4—C5	1.393 (5)	C18—H4A	0.9700
C4—C7	1.445 (5)	C18—H4B	0.9700
C5—C6	1.367 (5)	C19—O1	1.411 (5)
C5—H18	0.9300	C19—H19A	0.9600
C6—N1	1.332 (5)	C19—H19B	0.9600
C6—H17	0.9300	C19—H19C	0.9600
C7—C8	1.329 (5)	C20—O1	1.367 (4)
C7—H12	0.9300	C20—C21	1.377 (5)
C8—C9	1.452 (5)	C20—C25	1.383 (6)
C8—H11	0.9300	C21—C22	1.375 (5)
C9—C14	1.392 (5)	C21—H21	0.9300
C9—C10	1.395 (5)	C22—C23	1.372 (5)
C10—C11	1.381 (5)	C22—H22	0.9300
C10—H9	0.9300	C23—C24	1.379 (6)
C11—C12	1.399 (5)	C23—S1	1.779 (4)
C11—H6	0.9300	C24—C25	1.368 (6)
C12—N2	1.371 (5)	C24—H24	0.9300
C12—C13	1.409 (5)	C25—H25	0.9300
C13—C14	1.360 (5)	O2—S1	1.434 (4)
C13—H7	0.9300	O3—S1	1.418 (3)
C14—H8	0.9300	O4—S1	1.423 (3)
C15—N2	1.457 (5)	O5—H5A	0.8498
C15—C16	1.500 (6)	O5—H5B	0.8501
C15—H2A	0.9700		
			
N1—C1—H16A	109.5	C15—C16—H1C	109.5
N1—C1—H16B	109.5	H1A—C16—H1C	109.5
H16A—C1—H16B	109.5	H1B—C16—H1C	109.5
N1—C1—H16C	109.5	C18—C17—H3A	109.5
H16A—C1—H16C	109.5	C18—C17—H3B	109.5
H16B—C1—H16C	109.5	H3A—C17—H3B	109.5
N1—C2—C3	121.8 (4)	C18—C17—H3C	109.5
N1—C2—H15	119.1	H3A—C17—H3C	109.5
C3—C2—H15	119.1	H3B—C17—H3C	109.5
C2—C3—C4	120.6 (4)	N2—C18—C17	114.1 (4)
C2—C3—H14	119.7	N2—C18—H4A	108.7
C4—C3—H14	119.7	C17—C18—H4A	108.7
C5—C4—C3	115.8 (4)	N2—C18—H4B	108.7
C5—C4—C7	119.7 (4)	C17—C18—H4B	108.7
C3—C4—C7	124.5 (4)	H4A—C18—H4B	107.6
C6—C5—C4	121.7 (4)	O1—C19—H19A	109.5
C6—C5—H18	119.1	O1—C19—H19B	109.5
C4—C5—H18	119.1	H19A—C19—H19B	109.5
N1—C6—C5	120.3 (4)	O1—C19—H19C	109.5
N1—C6—H17	119.9	H19A—C19—H19C	109.5
C5—C6—H17	119.9	H19B—C19—H19C	109.5
C8—C7—C4	125.8 (4)	O1—C20—C21	115.6 (4)
C8—C7—H12	117.1	O1—C20—C25	124.2 (4)
C4—C7—H12	117.1	C21—C20—C25	120.1 (4)
C7—C8—C9	126.9 (4)	C22—C21—C20	119.8 (4)
C7—C8—H11	116.6	C22—C21—H21	120.1
C9—C8—H11	116.6	C20—C21—H21	120.1
C14—C9—C10	116.4 (4)	C23—C22—C21	120.8 (4)
C14—C9—C8	123.3 (4)	C23—C22—H22	119.6
C10—C9—C8	120.3 (4)	C21—C22—H22	119.6
C11—C10—C9	122.5 (4)	C22—C23—C24	118.8 (4)
C11—C10—H9	118.8	C22—C23—S1	121.3 (3)
C9—C10—H9	118.8	C24—C23—S1	119.9 (3)
C10—C11—C12	120.5 (4)	C25—C24—C23	121.5 (4)
C10—C11—H6	119.8	C25—C24—H24	119.3
C12—C11—H6	119.8	C23—C24—H24	119.3
N2—C12—C11	121.7 (4)	C24—C25—C20	119.1 (4)
N2—C12—C13	121.3 (4)	C24—C25—H25	120.5
C11—C12—C13	116.9 (4)	C20—C25—H25	120.5
C14—C13—C12	121.7 (4)	C6—N1—C2	119.9 (4)
C14—C13—H7	119.2	C6—N1—C1	120.3 (4)
C12—C13—H7	119.2	C2—N1—C1	119.9 (4)
C13—C14—C9	122.1 (4)	C12—N2—C15	121.1 (3)
C13—C14—H8	118.9	C12—N2—C18	121.6 (3)
C9—C14—H8	118.9	C15—N2—C18	116.6 (3)
N2—C15—C16	114.5 (4)	C20—O1—C19	118.6 (3)
N2—C15—H2A	108.6	H5A—O5—H5B	109.4
C16—C15—H2A	108.6	O3—S1—O4	113.3 (3)
N2—C15—H2B	108.6	O3—S1—O2	111.9 (2)
C16—C15—H2B	108.6	O4—S1—O2	113.0 (2)
H2A—C15—H2B	107.6	O3—S1—C23	105.6 (2)
C15—C16—H1A	109.5	O4—S1—C23	105.90 (19)
C15—C16—H1B	109.5	O2—S1—C23	106.5 (2)
H1A—C16—H1B	109.5		
			
N1—C2—C3—C4	−0.1 (7)	C22—C23—C24—C25	0.1 (7)
C2—C3—C4—C5	1.2 (7)	S1—C23—C24—C25	−179.0 (4)
C2—C3—C4—C7	−178.7 (4)	C23—C24—C25—C20	0.5 (7)
C3—C4—C5—C6	−1.9 (6)	O1—C20—C25—C24	179.9 (4)
C7—C4—C5—C6	178.0 (4)	C21—C20—C25—C24	−1.5 (7)
C4—C5—C6—N1	1.5 (7)	C5—C6—N1—C2	−0.2 (6)
C5—C4—C7—C8	−173.2 (4)	C5—C6—N1—C1	179.1 (4)
C3—C4—C7—C8	6.7 (7)	C3—C2—N1—C6	−0.5 (7)
C4—C7—C8—C9	178.7 (4)	C3—C2—N1—C1	−179.8 (4)
C7—C8—C9—C14	−0.9 (7)	C11—C12—N2—C15	13.1 (6)
C7—C8—C9—C10	177.3 (4)	C13—C12—N2—C15	−170.1 (4)
C14—C9—C10—C11	−0.2 (6)	C11—C12—N2—C18	−176.2 (4)
C8—C9—C10—C11	−178.5 (4)	C13—C12—N2—C18	0.6 (6)
C9—C10—C11—C12	1.1 (6)	C16—C15—N2—C12	77.0 (5)
C10—C11—C12—N2	175.9 (4)	C16—C15—N2—C18	−94.1 (5)
C10—C11—C12—C13	−1.1 (6)	C17—C18—N2—C12	−81.7 (5)
N2—C12—C13—C14	−176.7 (4)	C17—C18—N2—C15	89.4 (5)
C11—C12—C13—C14	0.3 (6)	C21—C20—O1—C19	−177.8 (4)
C12—C13—C14—C9	0.6 (6)	C25—C20—O1—C19	0.9 (6)
C10—C9—C14—C13	−0.6 (6)	C22—C23—S1—O3	108.6 (4)
C8—C9—C14—C13	177.6 (4)	C24—C23—S1—O3	−72.3 (4)
O1—C20—C21—C22	−179.3 (4)	C22—C23—S1—O4	−131.0 (4)
C25—C20—C21—C22	2.0 (6)	C24—C23—S1—O4	48.1 (4)
C20—C21—C22—C23	−1.3 (6)	C22—C23—S1—O2	−10.4 (4)
C21—C22—C23—C24	0.3 (6)	C24—C23—S1—O2	168.6 (4)
C21—C22—C23—S1	179.4 (3)		

### 4-{2-[4-(Diethylamino)phenyl]ethenyl}-1-methylpyridin-1-ium 4-methoxybenzene-1-sulfonate monohydrate (II) Hydrogen-bond geometry (Å, º)

**Table:**

*D*—H···*A*	*D*—H	H···*A*	*D*···*A*	*D*—H···*A*
O5—H5*A*···O3	0.85	2.06	2.891 (6)	165
O5—H5*B*···O3^i^	0.85	2.06	2.882 (6)	162
C3—H14···O5^ii^	0.93	2.49	3.394 (6)	163
C6—H17···O4^iii^	0.93	2.33	3.247 (6)	169
C7—H12···O2^iv^	0.93	2.58	3.476 (6)	162
C19—H19*A*···O2^ii^	0.96	2.53	3.423 (6)	155

Symmetry codes: (i) −*x*, −*y*+2, −*z*+1; (ii) *x*+1, *y*, *z*; (iii) −*x*+2, −*y*+1, −*z*+1; (iv) −*x*+1, −*y*+1, −*z*+1.
